# Pollution intensity-dependent metal accumulation in ground beetles: a meta-analysis

**DOI:** 10.1007/s11356-019-06294-5

**Published:** 2019-09-07

**Authors:** Dávid Tőzsér, Tibor Magura, Edina Simon, Szabolcs Mizser, Dalma Papp, Béla Tóthmérész

**Affiliations:** 1 Department of Ecology, University of Debrecen, Egyetem tér 1, Debrecen, H-4032 Hungary; 2 MTA-DE Biodiversity and Ecosystem Services Research Group, Egyetem tér 1, Debrecen, H-4032 Hungary

**Keywords:** Bioindicator, Carabid, Contamination, Detoxification mechanism, Soil pollution

## Abstract

Survival of organisms in polluted habitats is a key factor regarding their long-term population persistence. To avoid harmful physiological effects of pollutants’ accumulation in organisms, decontamination and excretion could be effective mechanisms. Among invertebrates, ground beetles are reliable indicators of environmental pollution. Published results, however, are inconsistent, as some studies showed effective decontamination and excretion of pollutants, while others demonstrated severe toxic symptoms due to extreme accumulation. Using ground beetles as model organisms, we tested our pollution intensity-dependent disposal hypothesis for five pollutants (Cd, Cu, Mn, Pb, and Zn) among four soil pollution intensity levels (low, moderate, high, and extreme) by categorical meta-analysis on published data. According to our hypothesis, decontamination and excretion of pollutants in ground beetles are effective in lowly or moderately polluted habitats, while disposal is ineffective in highly or extremely polluted ones, contributing to intense accumulation of pollutants in ground beetles. In accordance with the hypothesis, we found that in an extremely polluted habitat, accumulation of Cd and Pb in ground beetles was significantly higher than in lowly polluted ones. These findings may suggest the entomoremediation potential of ground beetles in an extremely polluted environment.

**Electronic supplementary material:**

The online version of this article (10.1007/s11356-019-06294-5) contains supplementary material, which is available to authorized users.

## Introduction

Habitat pollution poses a huge risk to organisms because of the direct and/or indirect contact with the contaminated environment (Ciadamidaro et al. 2017; Touceda-González et al. 2017). Among negative effects, restricted range and disappearance are common phenomena, while in the cases of certain species and intensities of pollution, survival could also be endangered (Acevedo-Whitehouse and Duffus 2009). Based on this, monitoring the fate of pollutants in ecosystems is highly important during environmental risk studies.

Responses of organisms to the metal pollution are greatly dependent on species and several other factors, such as form and concentration of metals, time of exposure, and pH conditions, which are closely related to the bioavailability of metals (Ashraf et al. 2012; Jaishankar et al. 2014; Rengel 2015). Furthermore, there are differences in the fate and regulation of essential (e.g., Cu, Mn, and Zn) and non-essential (e.g., Cd and Pb) metals in organisms, which is also a factor to consider (Hejna et al. 2018). Species that are able to indicate changes in environmental conditions like elevated metal concentrations are called bioindicators (Uehara-Prado et al. 2009; Parmar et al. 2016). This potential can manifest itself as the absence or presence of species, or as altered physiological and/or morphological characteristics (Bednarska et al. 2016). In metal-polluted environments, these possible responses are usually coupled with increased metal concentrations in tissues (Khan et al. 2015; Tőzsér et al. 2017; Papp et al. 2018; Mukhtorova et al. 2019).

Several studies have investigated the potential of terrestrial invertebrates to assess the degree of soil metal pollution (Mazzei et al. 2014; Xu et al. 2016a), among which ground beetles (Carabidae) are extensively studied indicator organisms (Rainio and Niemelä 2003; Pearce and Venier 2006; Rahim et al. 2013; Ghannem et al. 2018). Furthermore, they are potential candidates in entomoremediation (Ewuim 2013). Ground beetles are one of the most studied taxon due to their high general abundance in most habitats (Read et al. 1987; Magura et al. 2017) and diverse food preference (Kulkarni et al. 2017), where the latter results in nutrient and metal uptake from various sources (Purchart and Kula 2007). In addition, ground beetles are easy to collect, while the taxonomic history of species is well documented (Hůrka 1996; Lövei and Sunderland 1996). However, in terms of their metal uptake, assessment of beetles is quite contradictory. Besides experimental designs and methods used in individual publications, results on metal accumulation are further influenced by several factors such as feeding preference (carnivorous, herbivorous, and omnivorous), breeding type (spring breeder and autumn breeder), and developmental stage (egg, larva, pupa, and adult) as well as physiology and sex (Skalski et al. 2010; Simon et al. 2016). Furthermore, seasonal changes can also affect beetles’ body metal concentrations (Butovsky 2011).

Metal accumulation was widely investigated and found to be varied by ground beetle species, while the accumulation potential of a certain species was also assessed inconsistently in previous publications. In comparison with other taxa, Heikens et al. (2001) found that the general metal concentration in ground beetles was significantly lower than that in spiders. As a possible reason, Kramarz (1999) attributed the relatively low metal concentration in ground beetles to the efficient decontamination and excretion mechanism of the digestive system. Based on these abilities, Butovsky (2011) referred to ground beetles as insects with generally low metal accumulation potential. It was previously demonstrated by van Straalen and van Wensem (1986) that Cd and Pb were accumulated in ground beetles in much lower concentrations than Cu and Zn. Involving ten ground beetle species, Purchart and Kula (2007) observed significant interspecific differences in Cd, Cu, Mn, and Pb accumulation. Furthermore, studying 28 ground beetle species, Stepanov et al. (1987) found major differences in metal accumulation between *Carabus* and *Pterostichus* species.

In addition, responses of ground beetles to metal stress were reported to be also inconsistent among studies. Monitoring *Pterostichus oblongopunctatus* individuals, Bednarska et al. (2016) reported severe gut degeneration as a result of Cd, Ni, and Zn pollution; however, the contribution of these metals to the development of the symptom was different. Furthermore, in the case of Zn pollution, Kramarz and Laskowski (1997) found a decrease in egg numbers laid by *Poecilus cupreus*. In contrast, in the case of *P. oblongopunctatus*, Lagisz and Laskowski (2008) observed increased egg production and decreased egg quality (hatchability) in metal-polluted areas compared to unpolluted ones. Lagisz et al. (2005) found that ground beetle species did not develop a successful adaptation mechanism to the toxic environmental conditions in metal-polluted habitats. In contrast, Zygmunt et al. (2006) demonstrated higher body mass of *P. oblongopunctatus* in the polluted areas than in an area with lower Cd, Pb, and Zn concentrations. It was explained by advanced metal tolerance and altered interspecific competition characteristics.

In the present study, ground beetles are used as model organisms to test our pollution intensity-dependent disposal hypothesis for five pollutants (Cd, Cu, Mn, Pb, and Zn) among four soil pollution intensity levels (low, moderate, high, and extreme) by categorical meta-analysis on published data. Studied metals were involved in this study due to their major importance in various environmental processes, and due to being the only metals with sufficient data amount for these analyses. We hypothesized that inconsistencies in published results concerning decontamination, excretion, and accumulation of pollutants in ground beetles should have arisen from various pollution intensities of habitats. Based on our pollution intensity-dependent disposal hypothesis, we assumed that decontamination and excretion of pollutants in ground beetles are effective in lowly or moderately polluted habitats, while disposal is ineffective in highly or extremely polluted ones, contributing to intense accumulation of pollutants in ground beetles.

## Materials and methods

### Literature search and data selection

Data for the meta-analysis were collected by a literature search on Web of Science for the period 1975–2018. The following search terms were used: TOPIC = (metal) AND TOPIC = (accumulat* OR stor* OR accru* OR collect* OR aggregat* OR accret* OR buil* OR grow* OR inflat* OR add*) AND TOPIC = (carabid* OR ground beetle*). For further, relevant publications, we revised the references of publications resulted in the search. To be suitable for the meta-analysis, publications had to publish metal (Cd, Cu, Mn, Pb, and Zn) concentrations (± SD with sample sizes) in one or more ground beetle species reared/found in unpolluted (control) vs. polluted habitats. Habitat pollution levels were determined by soil pollution levels; thus, publications had to report data on soil metal concentrations. Studies in which ground beetles had been fed contaminated food were excluded from the analyses. To get a comprehensive view of the metal accumulation potential of ground beetles, studied species were analyzed collectively.

### Statistical analyses

For each unpolluted-to-polluted comparison, a common effect size, the unbiased standardized mean difference (Hedges’ *g*), was calculated between unpolluted and polluted ground beetle groups:

1 $$ g=J\frac{\overline{X_{\mathrm{U}}}-\overline{X_{\mathrm{P}}}}{S_{\mathrm{within}}} $$

2 $$ {S}_{\mathrm{within}}=\sqrt{\frac{\left({n}_{\mathrm{U}}-1\right){S}_{\mathrm{U}}^2+\left({n}_{\mathrm{P}}-1\right){S}_{\mathrm{P}}^2}{n_{\mathrm{U}}+{n}_{\mathrm{P}}-2}} $$

3 $$ J=1-\frac{3}{4\left({n}_{\mathrm{U}}+{n}_{\mathrm{P}}-2\right)-1}, $$

where $\overline{X_{\mathrm{U}}}$ and $\overline{X_{\mathrm{P}}}$ are the mean metal concentrations (mg kg^−1^, dry matter) in ground beetle species reared/found in unpolluted (U) and polluted (P) habitats, *n*_U_ and *n*_P_ are the sample sizes of ground beetles from unpolluted (U) and polluted (P) habitats, and *S*_U_ and *S*_P_ are their standard deviations. A negative *g* value refers to higher metal concentration in ground beetles from polluted than from unpolluted soil.

We used subgroup meta-analysis to determine whether metal accumulation was similar among the differently polluted habitats. The subgroups were the habitats with different pollution intensity levels. Pollution levels were determined by the calculation of the pollution index (PI), which refers to the ratio of the detected and the background metal concentration in soils (Faiz et al. 2009):

4 $$ \mathrm{PI}=\frac{M_{\mathrm{c}}}{B_{\mathrm{c}}}, $$

where *M*_c_ is the measured metal concentration in soil (reported in the given paper) and *B*_c_ is the background metal concentration in soil (mg kg^−1^, dry matter). Based on this calculation, pollution intensity levels were determined by the following: PI ≤ 1 (low), 1 ≤ PI ≤ 2 (moderate), 2 ≤ PI ≤ 5 (high), and PI ≥ 5 (extreme) (Lu et al. 2007; Simon et al. 2013). Background metal concentrations were used from Geochemical Atlas of Europe Part 1 (Salminen et al. 2006).

We estimated the overall effect and examined the effects of moderators (pollution levels) using a random-effects model. We used a random-effects model because studies were not expected to estimate a common effect size due to variability in locations, habitats, and other conditions and methods used in the individual studies (Borenstein et al. 2009). Random-effects models are more plausible than fixed-effect ones and also attribute the distribution of effect sizes to real differences among studies and do not assume sampling error as the only source of differences in effect sizes between studies (Borenstein et al. 2009). The mean effect size was defined as statistically significant if the 95% bootstrap confidence interval (CI; calculated with 999 iterations) did not include zero.

We investigated whether effect sizes were homogenous or varied across studies (i.e., if there was heterogeneity), since if the effect sizes vary across studies, a fundamentally different interpretation is needed compared to consistent effect sizes. To assess the heterogeneity of effects between studies, complementary measures of heterogeneity, *Q*, *T*^2^, and *I*^2^, were calculated (Borenstein et al. 2009). Further, we partitioned the total variance (*Q*_total_) into within-group (*Q*_within_) and between-group (*Q*_between_) variances using a *Q*-test based on analysis of variance. Then, these different components of variance were tested for statistical significance (Borenstein et al. 2009). In the case of significant variance between groups (*Q*_between_), metal accumulation of beetles from polluted habitats (soils) was significantly different according to the pollution intensity of the habitat. In order to evaluate the proportion of true variance explained by the covariates (subgroup classification), the *R*^2^ was calculated (Borenstein et al. 2009). During calculations, subgroups with less than three cases were excluded from subgroup analyses, if a restricted amount of data was presented.

In meta-analyses, publication bias resulting in missing studies and potentially biased effect sizes are frequent issues (Borenstein et al. 2009). Hence, we tested publication bias by using funnel plots and Egger’s test (Borenstein et al. 2009). By significant asymmetry, we used the trim-and-fill method (Duval and Tweedie 2000). This method calculates the number of missing studies and estimates their effect sizes as well as standard errors. After this, the resulted missing studies are added to the data set of the meta-analysis, and the summary effect size is re-computed. This method yields an unbiased estimate of the summary effect size (Borenstein et al. 2009). Meta-analyses, heterogeneity measures, and assessment of publication bias were completed by the *MAd* and *metafor* packages (Viechtbauer 2010; Del Re and Hoyt 2014) operated in the R version 3.5.0 (R Core Team 2018).

## Results

### Literature search and data selection

The literature search yielded 72 publications, out of which, after checking also their reference sections, six papers were found that reported metal (Cd, Cu, Mn, Pb, and Zn) concentrations (± SD with sample sizes) in one or more ground beetle species from both unpolluted (control) and polluted habitats (soils) (Supplementary Materials Table A.1). From applicable publications, 146 comparisons were recovered. In these papers, 10 carnivorous ground beetle species were studied (Supplementary Materials Table A.1). In the six papers, soil metal concentrations varied between wide ranges: 0.24–81.9 mg kg^−1^ for Cd, 6.5–46.9 mg kg^−1^ for Cu, 309–5827 mg kg^−1^ for Mn, 54.8–2635 mg kg^−1^ for Pb, and 22.5–10454 mg kg^−1^ for Zn. Based on pollution index (PI) calculations, data were available from habitats with low and extreme pollution intensities for Mn and Pb; moderate and extreme pollution intensities for Cd; low, high, and extreme pollution intensities for Zn; and low, moderate, high, and extreme pollution intensities for Cu.

### Metal accumulation in beetles

#### Accumulation of Cd

**Fig. 1:**
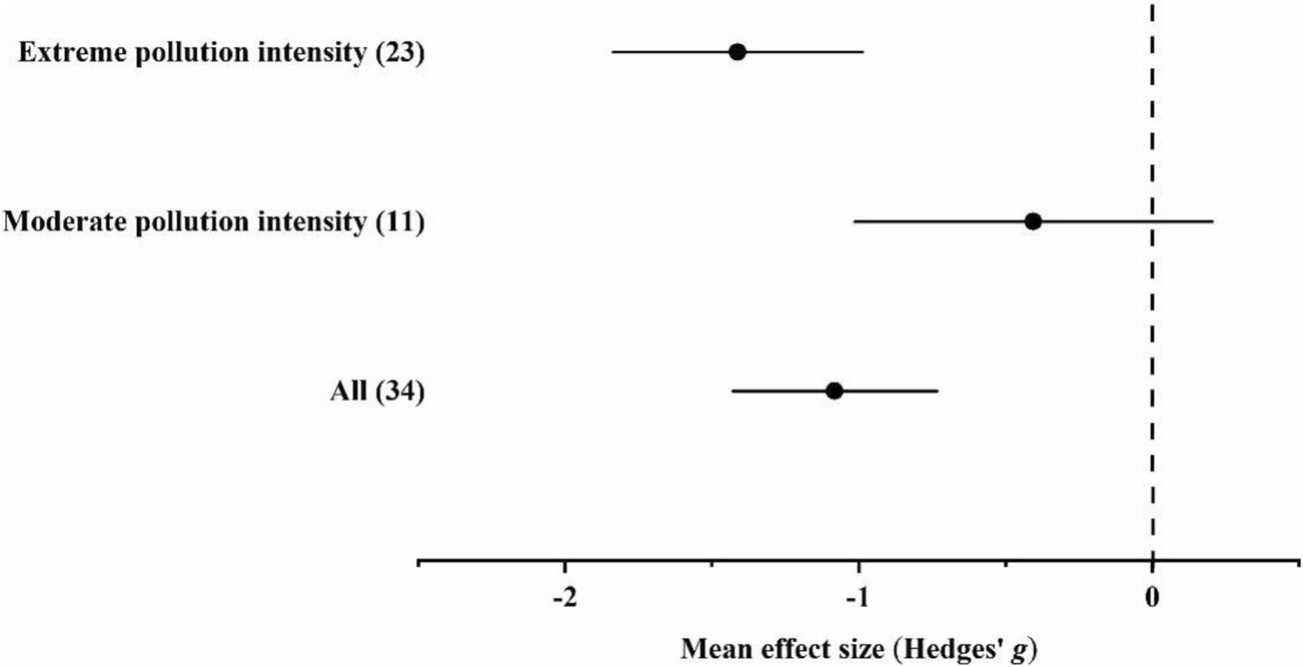
Mean effect sizes (mean Hedges’ *g* ± 95% confidence interval) for Cd concentrations in ground beetle individuals living in unpolluted and polluted habitats. Values in brackets refer to the number of comparisons from which the mean effect size was calculated. A negative *g* value means higher Cd concentration in beetles living in polluted habitats than in unpolluted ones. The mean effect size was considered statistically significant if the 95% bootstrap confidence interval (CI) did not include zero

In the case of Cd, data were available only from habitats with moderate and extreme pollution intensities. In extremely polluted habitats, ground beetles accumulated Cd in significantly (*p* < 0.05) higher concentrations than individuals in unpolluted habitats. The general Cd accumulation potential of ground beetles was significantly higher in polluted than in unpolluted habitats (Fig. 1 and Supplementary Materials Tables B.1–2).

In the overall model, total heterogeneity was significant and significant residual, unexplained heterogeneity was also found (Supplementary Materials Table B.1–2). In the funnel plot, significant asymmetry was found by the random-effects version of Egger’s test, while it was not found by the classical version. In addition, according to the trim-and-fill method, the estimated number of missing values was 0 (Supplementary Materials C.1).

#### Accumulation of Cu

**Fig. 2:**
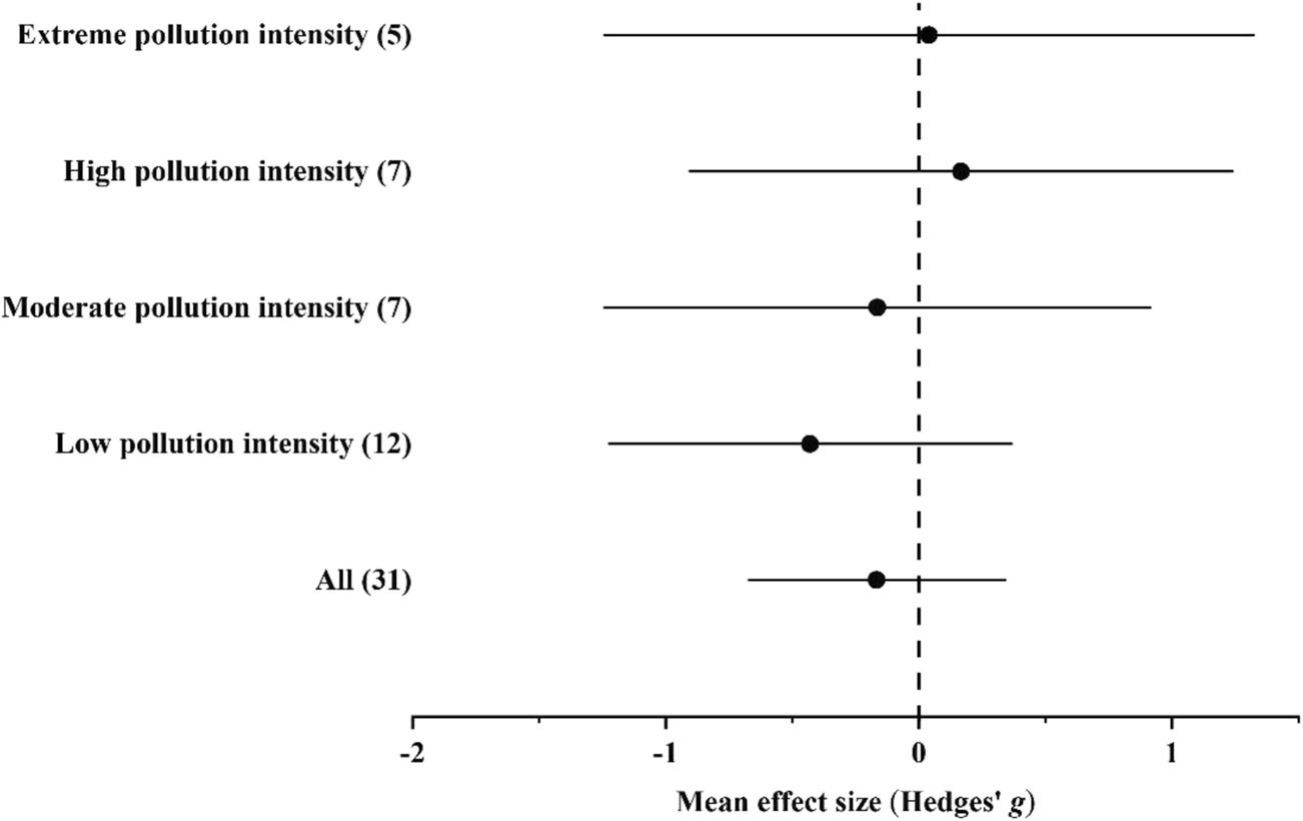
Mean effect sizes (mean Hedges’ *g* ± 95% confidence interval) for Cu concentrations in ground beetle individuals living in unpolluted and polluted habitats. Values in brackets refer to the number of comparisons from which the mean effect size was calculated. A negative *g* value means higher Cu concentration in beetles living in polluted habitats than in unpolluted ones. The mean effect size was considered statistically significant if the 95% bootstrap confidence interval (CI) did not include zero

In the case of Cu, data were available from habitats with low, moderate, high, and extreme pollution intensities. Ground beetles accumulated Cu in higher concentrations in polluted habitats than in unpolluted ones; however, the differences were insignificant by either of the pollution intensity levels (Fig. 2 and Supplementary Materials Tables B.3–4).

In the overall model, total heterogeneity was significant and significant residual, unexplained heterogeneity was also found (Supplementary Materials Table B.3–4). In the funnel plot, significant asymmetry was found by the random-effects version of Egger’s test, while it was not found by the classical version. In addition, according to the trim-and-fill method, the estimated number of missing values was 0 (Supplementary Materials C.2).

#### Accumulation of Mn

**Fig. 3:**
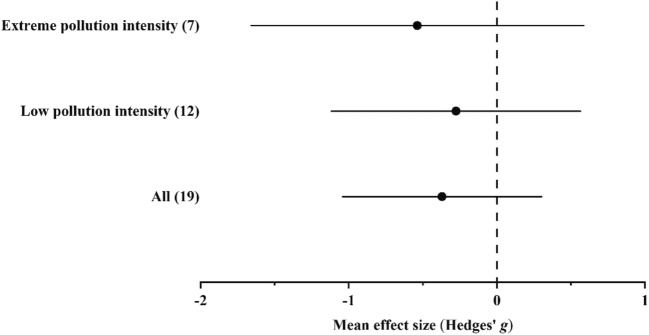
Mean effect sizes (mean Hedges’ *g* ± 95% confidence interval) for Mn concentrations in ground beetle individuals living in unpolluted and polluted habitats. Values in brackets refer to the number of comparisons from which the mean effect size was calculated. A negative *g* value means higher Mn concentration in beetles living in polluted habitats than in unpolluted ones. The mean effect size was considered statistically significant if the 95% bootstrap confidence interval (CI) did not include zero

In the case of Mn, data were available only from habitats with low and extreme pollution intensities. Ground beetles accumulated Mn in higher concentrations in polluted habitats than in unpolluted ones; however, differences were insignificant by either of the pollution intensity levels (Fig. 3 and Supplementary Materials Tables B.5–6).

In the overall model, total heterogeneity was significant and significant residual, unexplained heterogeneity was also found (Supplementary Materials Table B.5–6). In the funnel plot, neither the random-effects version nor the classical version of Egger’s test showed significant asymmetry. In addition, according to the trim-and-fill method, the estimated number of missing values was 0 (Supplementary Materials C.3).

#### Accumulation of Pb

**Fig. 4:**
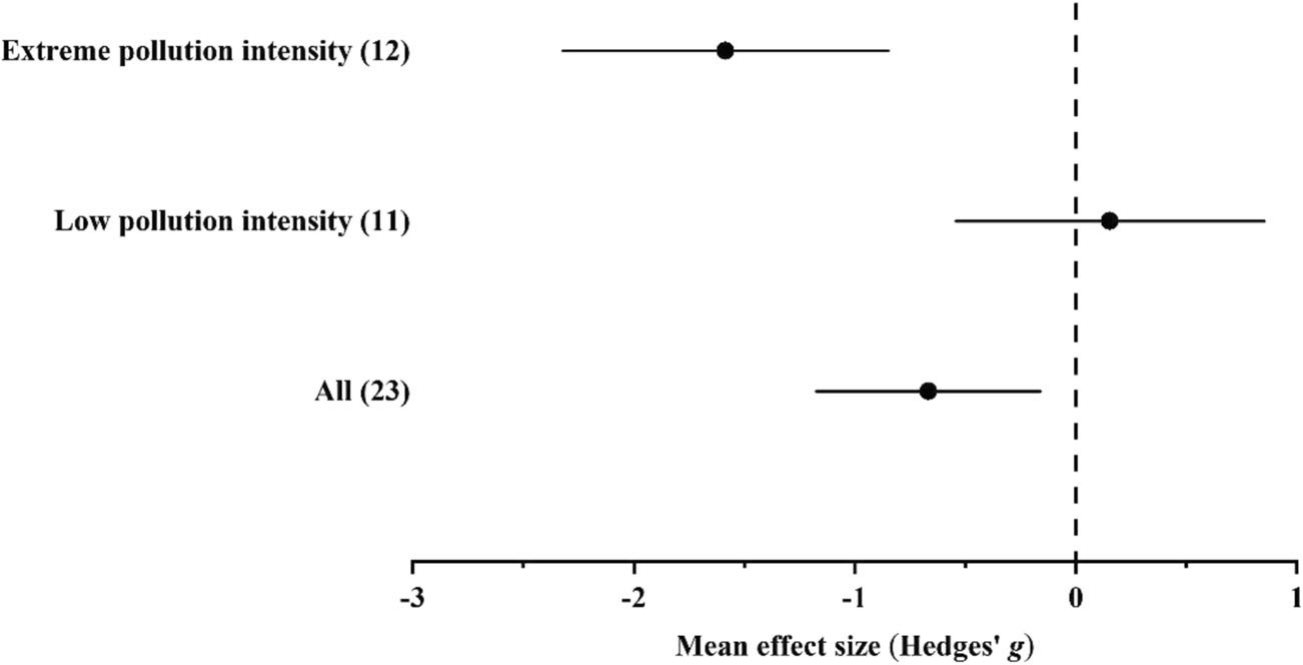
Mean effect sizes (mean Hedges’ *g* ± 95% confidence interval) for Pb concentrations in ground beetle individuals living in unpolluted and polluted habitats. Values in brackets refer to the number of comparisons from which the mean effect size was calculated. A negative *g* value means higher Pb concentration in beetles living in polluted habitats than in unpolluted ones. The mean effect size was considered statistically significant if the 95% bootstrap confidence interval (CI) did not include zero

In the case of Pb, data were available only from habitats with low and extreme pollution intensities. In extremely polluted habitats, ground beetles accumulated Pb in significantly (*p* < 0.05) higher concentrations than individuals in unpolluted habitats. The general Pb accumulation potential of ground beetles was significantly higher in polluted than in unpolluted habitats (Fig. 4 and Supplementary Materials Tables B.7–8).

In the overall model, total heterogeneity was significant and significant residual, unexplained heterogeneity was also found (Supplementary Materials Table B.7–8). In the funnel plot, significant asymmetry was found both by the random-effects and classical version of Egger’s test. In addition, according to the trim-and-fill method, the estimated number of missing values was 0 (Supplementary Materials C.1).

#### Accumulation of Zn

**Fig. 5:**
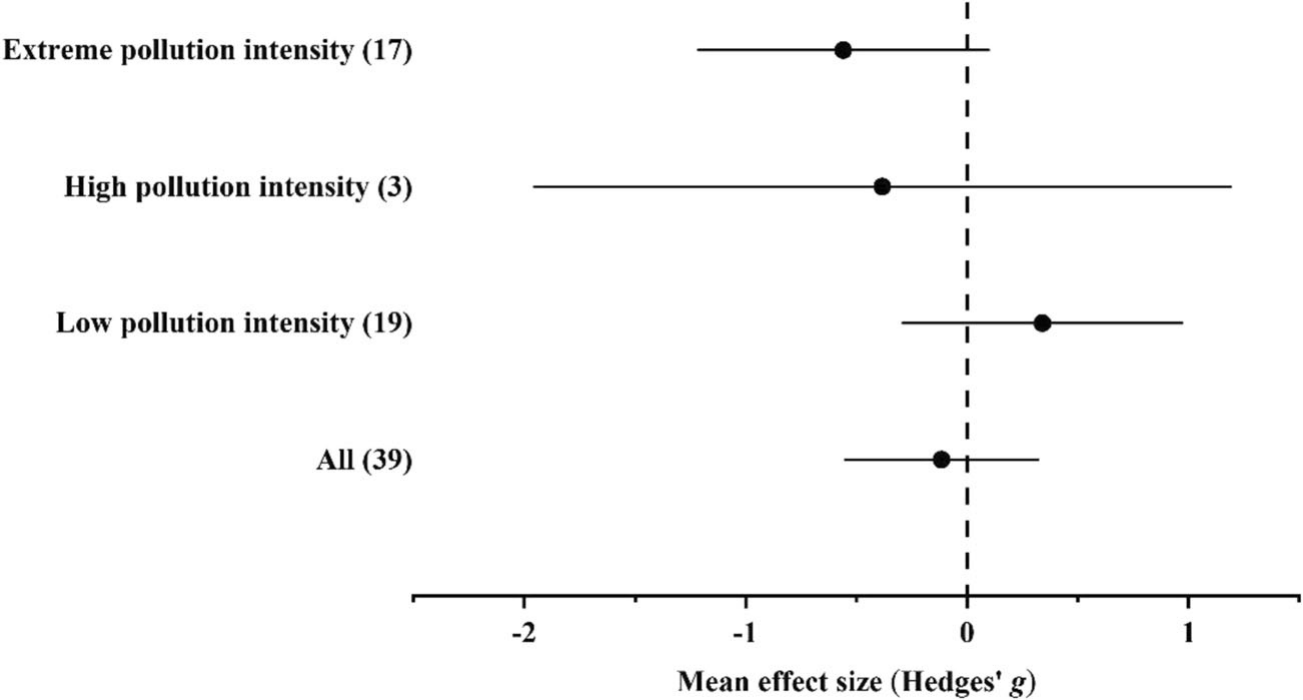
Mean effect sizes (mean Hedges’ *g* ± 95% confidence interval) for Zn concentrations in ground beetle individuals living in unpolluted and polluted habitats. Values in brackets refer to the number of comparisons from which the mean effect size was calculated. A negative *g* value means higher Zn concentration in beetles living in polluted habitats than in unpolluted ones. The mean effect size was considered statistically significant if the 95% bootstrap confidence interval (CI) did not include zero

In the case of Zn, data were available from habitats with low, high, and extreme pollution intensities. Ground beetles accumulated Zn in higher concentrations in polluted habitats than in unpolluted ones; however, the differences were insignificant by either of the pollution intensity levels (Fig. 5 and Supplementary Materials Tables B.9–10).

In the overall model, total heterogeneity was significant and significant residual, unexplained heterogeneity was also found (Supplementary Materials Table B.9–10). In the funnel plot, significant asymmetry was found by the random-effects version of Egger’s test, while it was not found by the classical version. In addition, according to the trim-and-fill method, the estimated number of missing values was 0 (Supplementary Materials C.2).

## Discussion

In line with our hypothesis, we demonstrated that ground beetles should have an effective detoxification mechanism by low soil Cd, Pb, and Zn pollution intensity, because we found low metal accumulation in their tissues. However, our result also indicated that the mechanism may be significantly inhibited by an extreme level of soil pollution.

In comparison with control environmental conditions, we found that ground beetles living in extremely polluted habitats accumulated Cd in significantly higher concentrations than those from habitats with low pollution intensity. A high concentration of Cd in ground beetles under highly toxic conditions was also found by Kramarz (1999). The author demonstrated that ground beetles, which were exposed to Cd-contaminated food, showed continuously increasing body metal concentration until the end of exposure. Then, due to the efficient inherent decontamination mechanism, Cd concentration rapidly decreased to pre-treatment levels. In contrast, Maryański et al. (2002) found that ground beetles were able to regulate excess Cd uptake with only a moderate level of decontamination efficiency. They concluded that the energy demand of the detoxification mechanism was high enough to influence reproduction success significantly. As a further consequence, Lindquist and Block (2001) observed that ground beetles living in the highly metal-polluted environment had much lower body fat concentration than individuals from unpolluted habitats, which was explained by the increased energy demand of metal detoxification. Similarly, Butovsky (1997) highlighted the commonly low accumulation propensity of Cd in ground beetles. Furthermore, Lodenius et al. (2009) noticed that compared to control (unpolluted) conditions, the Cd concentration in beetles remained low even after soil fertilization and the simultaneous increase in the amount of available Cd. Also, in accordance with our observations, Purchart and Kula (2007) demonstrated a generally low Cd accumulation potential in several ground beetle species collected from a habitat with a low pollution level. Thus, it was possible in extremely polluted habitats that continuous Cd exposure, coupled with restricted detoxification success, could contribute to the high body concentration found in ground beetles. In habitats with low pollution intensity, reduced accumulation and increased detoxification (excretion) intensity (Kramarz 1999) may explain low body Cd concentrations presented in this paper.

We observed that accumulation of Pb in ground beetles was considerable in extremely polluted habitats. In contrast with our findings, Butovsky (1997) concluded that Pb and Cd have the lowest accumulation potential in ground beetle species. In the cases of several terrestrial invertebrates, Didur et al. (2017) also demonstrated that the studied species had generally low Pb accumulation rates, regardless of their position in the trophic chain. As presented in this paper, Zhang et al. (2017) also found increased Pb concentration in *Enchytraeus crypticus* individuals inhabiting heavily polluted habitats. They emphasized that after the 14-day accumulation period, individuals could regulate body metal concentration during an elimination period and decrease it to a constant low level with their successful excretion mechanism. The successful decontamination ability of ground beetles is based on the low pollution intensity and restricted availability of Pb in soils along with the low Pb accumulation. As for soil conditions, Pb is a metal with very low mobility; however, its mobility depends on such soil properties as pH (Ashraf et al. 2012). Based on this, Pb mobility could be increased by acidic soil pH values (Jelaska et al. 2007). Thus, high mobility of Pb could explain the significantly higher Pb concentration in ground beetles. This result is in correspondence with the observations of Heikens et al. (2001), who found a positive correlation between soil and invertebrate body metal concentrations in heavily polluted habitats. Van Straalen et al. (2001) also highlighted the notable Pb-accumulating potential of ground beetles in extremely polluted habitats. As a conclusion, it could be recognized that Pb accumulation is dependent on several factors and is greatly variable among studies.

In our study, we found the highest degree of Zn accumulation in ground beetle species in extremely polluted conditions. It was observed by Mozdzer et al. (2003) that Zn pollution induced developmental abnormalities in *P. oblongopunctatus* individuals similar to those caused by Cd. However, Grodzinska et al. (1987) demonstrated that excretion of Zn in terrestrial species can be more successfully regulated than that of non-essential metals. In addition, Kramarz (1999) revealed a significant effect of Cd-Zn co-contamination neither on Cd nor on Zn accumulation in ground beetles, indicating a certain degree of correlation between the accumulation mechanisms of these two metals. This latter relation is in accordance with our results, regarding Cd and Zn accumulations in extremely polluted habitats. Similar to our findings, Gongalski and Butovsky (1998) observed no significant difference in Zn accumulation of *Poecilus cupreus* between highly polluted and unpolluted soils. Low Zn concentrations in beetles in slightly polluted habitats could be the result of several factors. For instance, Lock et al. (2001) found that ground beetles collected from differently polluted habitats could have low body Zn concentration due to the efficient regulatory and decontamination mechanism of their preys. In line with that, we assume that carnivorous species involved in these analyses had relatively high Zn accumulation potential, compared to non-studied omnivore species. This assumption was previously confirmed by Purchart and Kula (2007), who highlighted that Zn uptake is more intensive in carnivorous than in herbivorous and omnivorous species.

Unlike Cd, Pb, and Zn, we found insignificant differences in Cu accumulation of ground beetles between the polluted and unpolluted habitats. An insignificant trend with generally higher *g* values for Cu from low towards extreme pollution intensity levels may indicate a decreasing accumulation intensity in beetles. However, the accumulation pattern of Cu depends basically on the mass/size of the ground beetles, with a lower accumulation rate in large species than in small-sized ones (Butovsky 2011). In our study, concentrations of Cu were calculated for both medium-sized and large-sized ground beetles, probably causing a different trend in accumulation intensity of Cu compared to those of Cd, Pb, and Zn. Moreover, for invertebrates, Lukáň (2009) emphasized that Cu is an essential element with special accumulation and regulation patterns and has higher body element concentration compared to non-essential metals like Cd. In contrast with our results, Lukáň (2009) indicated a general positive correlation between soil and body Cu concentrations. In addition, Talarico et al. (2014) also observed a close relationship in Cu concentration between soil and large-sized *Carabus lefebvrei* individuals. However, Smolders et al. (2012) highlighted that the availability of Cu in soils could be highly variable depending on the form and exposure of the metal. In association with these results, Bednarska and Stępień (2015) found that red flour beetles (*Tribolium castaneum*) responded to a considerably elevated soil Cu concentration only with slightly increased body concentration. The authors attributed this phenomenon to the efficient internal regulation route, as previously indicated by Lukáň (2009). In white rat springtail (*Folsomia candida*) individuals, Ardestani and van Gestel (2013) found similar results between habitats with high and low Cu pollution. Based on these results, we assume that insignificant differences in ground beetle body Cu concentrations could be related to the mass/size and detoxification mechanism of the studied species, complemented by a habitat-specific availability of soil Cu.

It was presented in this study that ground beetles did not accumulate significantly higher concentrations of Mn in polluted habitats than in unpolluted ones. Furthermore, studying the effect of Mn accumulation on the uptake of other metals in ground beetles, Purchart and Kula (2007) demonstrated a significant positive correlation between the accumulations of Mn and Cu. We indicated that the two metals had similar accumulation patterns in the studied ground beetle species. Between the accumulations of Mn and Cd in terrestrial organisms, Huang et al. (2017) found a negative correlation. Similarly, we observed that ground beetles had high Cd and low Mn accumulation potentials in habitats with high pollution intensity. Knowing that each of the species involved in this meta-analysis is carnivorous, metal concentrations in individuals could arise from consuming preys feeding on plants with the contrasting Mn-Cd accumulation pattern. Thus, indirect uptake of metals could be realized through the food chain by the biomagnification process (Conti et al. 2017). Investigating metal accumulation in several arthropod species, Janssen and Hogervorst (1993) found no major differences in Mn concentration of species collected from polluted and reference areas. It was previously indicated that despite being present in soils in sufficient concentrations, Mn availability to plants could be highly reduced by certain soil and plant characteristics (e.g., alteration of soil pH, amount and quality root exudates; Rengel 2015). In contrast, Jelaska et al. (2007) found significant differences in soil pH between polluted and unpolluted habitats, while ground beetles did not respond to these altered environmental conditions with an increased rate of Mn accumulation. We assume that this kind of specific accumulation mechanism of essential Mn could be responsible for the limited concentration values in beetles.

Based on the meta-analysis, significant residual and unexplained heterogeneities were found, which indicated that besides pollution intensity levels there are several other factors that should be considered when assessing metal accumulation in ground beetles. Partition between available and total metal concentrations, and forms of metals in soils, are decisive respecting the accumulation in beetles and also in other organisms (Ignatowicz 2017). Additionally, other soil parameters such as soil pH, loam content, and hydrological conditions also influence the migration and uptake of metals considerably (Rakesh Sharma and Raju 2013; Xu et al. 2016b). In addition, ground beetle species and their specific inherent characteristics determine the accumulation pattern significantly (Avgın and Luff 2010; Butovsky 2011). It was previously demonstrated that body size (Butovsky 2011), sex (Stepanov et al. 1987; Rabitsch 1995; Lagisz et al. 2010), feeding preference (Migula et al. 2004; Purchart and Kula 2007), breeding type (Skalski et al. 2010), and developmental stage (Bayley et al. 1995; Bednarska et al. 2011) are of great importance regarding metal uptake in beetles. Exposure time is also a major factor to consider (Spurgeon and Hopkin 1999; Prasifka et al. 2008). A comprehensive meta-analysis involving all the above factors influencing metal uptake could be a major step towards more thoroughly assessing of the metal accumulation pattern in ground beetles.

## Conclusions

We found that metal accumulation of ground beetles was greatly variable depending on the studied metal and pollution intensity level. In habitats with a low pollution level, ground beetles can regulate their body metal concentration via a successful detoxification mechanism. In extremely polluted habitats, ground beetles showed a significant accumulation potential for Cd and Pb, and a great accumulation potential for Zn; thus, ground beetles were found to be good indicators of extreme soil metal pollution via metal uptake. Summarizing, our results suggest that ground beetles can effectively be used as a naturally available pollution indicator or bioassays of the level of soil pollution. Furthermore, ground beetles as entomoremediators may be useful in decontaminating soils extremely polluted by metals via sequestering metals in their tissues (Ewuim 2013).

## Electronic supplementary material

ESM 1 (DOCX 434 kb)
