# Supplementary material for: Pollution intensity-dependent metal accumulation in ground beetles: a meta-analysis
Source: Environ Sci Pollut Res Int. 2019 Sep 7;26(31):32092–102. doi: 10.1007/s11356-019-06294-5 (PMC6875149; doi:10.1007/s11356-019-06294-5)
Supplement: Supplementary file 1 — (DOCX 434 kb) [file 11356_2019_6294_MOESM1_ESM.docx]

**Supplementary Materials Table A.1** Publications used in the meta-analysis, which reported mean values of metal concentrations, standard deviation, and sample size for ground beetle species from uncontaminated and uncontaminated habitats. (Reference numbers are in superscription; see bibliographic data of the papers below)

| **Publication** | **Studied species** | **Studied metals** | **Number of**  **comparisons** |
| --- | --- | --- | --- |
| Bednarska et al. 2013 ^1^ | *Pterostichus oblongopunctatus* | Cd, Zn | 16 |
| Jelaska et al. 2007 ^2^ | *Abax parallelepipedus*  *Carabus convexus*  *Carabus coriaceus*  *Carabus intricatus*  *Carabus nemoralis*  *Carabus ulrichii*  *Carabus violaceus* | Cd, Cu, Mn, Pb, Zn | 55 |
| Lodenius et al. 2009 ^3^ | *Carabus glabratus*  *Pterostichus niger* | Cd | 3 |
| Migula et al. 2004 ^4^ | *Pterostichus oblongopunctatus* | Cd, Cu, Pb, Zn | 16 |
| Simon et al. 2016 ^5^ | *Carabus violaceus*  *Pterostichus oblongopunctatus* | Cd, Cu, Mn, Zn | 24 |
| Stone et al. 2002 ^6^ | *Pterostichus oblongopunctatus* | Cd, Cu, Pb, Zn | 32 |

**Bibliographic data of the papers**

^1^ Bednarska AJ, Stachowicz I, Kuriańska L (2013) Energy reserves and accumulation of metals in the ground beetle *Pterostichus oblongopunctatus* from two metal-polluted gradients. Environ Sci Pollut R 20:390–398. https://doi.org/10.1007/s11356-012-0993-y

^2^ Jelaska LS, Blanuša M, Durbešić P, Jelaska SD (2007) Heavy metal concentrations in ground beetles, leaf litter, and soil of a forest ecosystem. Ecotox Environ Safe 66:74–81. https://doi.org/10.1016/j.ecoenv.2005.10.017

^3^ Lodenius M, Josefsson J, Heliövaara K, Tulisalo E, Nummelin M (2009) Cadmium in insects after ash fertilization. Insect Sci 16:93–98. https://doi.org/10.1111/j.1744-7917.2009.00259.x

^4^ Migula P, Łaszczyca P, Augustyniak M, Wilczek G, Pozpędek K, Kafel A, Wołoszyn M (2004) Antioxidative defence enzymes in beetles from a metal pollution gradient. Biologia 59:645–654.

^5^ Simon E, Harangi S, Baranyai E, Braun M, Fábián I, Mizser Sz, Nagy L, Tóthmérész, B (2016) Distribution of toxic elements between biotic and abiotic components of terrestrial ecosystem along an urbanization gradient: Soil, leaf litter and ground beetles. Ecol Indic 60:258–264. https://doi.org/10.1016/j.ecolind.2015.06.045

^6^ Stone D, Jepson P, Laskowski R (2002) Trends in detoxification enzymes and heavy metal accumulation in ground beetles (Coleoptera: Carabidae) inhabiting a gradient of pollution. Comp Biochem Phys C 132:105–112. https://doi.org/10.1016/S1532-0456(02)00052-2

**Supplementary Materials Table B.1–2** Estimates and heterogeneities in the models for Cd concentrations in ground beetle individuals living in uncontaminated and contaminated habitats

| **Subgroup** | **Mean effect size (model)** | **Lower CI bound (model)** | **Upper CI bound (model)** | **SE (model)** | ***p* value (model)** | **Q (hetero-geneity)** | ***p* value (hetero-geneity)** | **Tau^2^** | **I^2^** | **R^2^** |
| --- | --- | --- | --- | --- | --- | --- | --- | --- | --- | --- |
| Moderate pollution intensity | -0.405 | -1.014 | 0.205 | 0.311 | 0.193 | 66.893 | < 0.001 | 1.187 | 85% |  |
| Extreme pollution intensity | -1.413 | -1.839 | -0.986 | 0.217 | < 0.001 | 125.997 | < 0.001 | 0.765 | 83% |  |
| Overall | -1.082 | -1.431 | -0.732 | 0.178 | < 0.001 | 276.078 | < 0.001 | 0.895 | 88% | 21.01% |

| **Component of variance** | ***Q*** | ***d.f.*** | ***p*** |
| --- | --- | --- | --- |
| Moderate pollution intensity | 66.893 | 10 | < 0.001 |
| Extreme pollution intensity | 125.997 | 22 | < 0.001 |
| Within | 192.078 | 32 | < 0.001 |
| Between | 7.056 | 1 | 0.008 |
| Total | 276.078 | 33 | < 0.001 |

**Supplementary Materials Table B.3–4** Estimates and heterogeneities in the models for Cu concentrations in ground beetle individuals living in uncontaminated and contaminated habitats

| **Subgroup** | **Mean effect size (model)** | **Lower CI bound (model)** | **Upper CI bound (model)** | **SE (model)** | ***p* value (model)** | **Q (hetero-geneity)** | ***p* value (hetero-geneity)** | **Tau^2^** | **I^2^** | **R^2^** |
| --- | --- | --- | --- | --- | --- | --- | --- | --- | --- | --- |
| Low pollution intensity | -0.429 | -1.226 | 0.367 | 0.406 | 0.291 | 185.236 | < 0.001 | 3.835 | 94% |  |
| Moderate pollution intensity | -0.164 | -1.244 | 0.915 | 0.551 | 0.766 | 61.707 | < 0.001 | 3.947 | 90% |  |
| High pollution intensity | 0.167 | -0.907 | 1.240 | 0.548 | 0.761 | 12.567 | 0.050 | 0.344 | 52% |  |
| Extreme pollution intensity | 0.041 | -1.243 | 1.324 | 0.655 | 0.951 | 15.195 | 0.004 | 1.062 | 74% |  |
| Overall | -0.166 | -0.672 | 0.340 | 0.258 | 0.521 | 280.849 | < 0.001 | 2.378 | 89% | 0% |

| **Component of variance** | ***Q*** | ***d.f.*** | ***p*** |
| --- | --- | --- | --- |
| Low pollution intensity | 185.236 | 11 | < 0.001 |
| Moderate pollution intensity | 61.707 | 6 | < 0.001 |
| High pollution intensity | 12.568 | 6 | 0.050 |
| Extreme pollution intensity | 15.195 | 4 | 0.004 |
| Within | 274.705 | 27 | < 0.001 |
| Between | 0.888 | 3 | 0.828 |
| Total | 280.849 | 30 | < 0.001 |

**Supplementary Materials Table B.5–6** Estimates and heterogeneities in the models for Mn concentrations in ground beetle individuals living in uncontaminated and contaminated habitats

| **Subgroup** | **Mean effect size (model)** | **Lower CI bound (model)** | **Upper CI bound (model)** | **SE (model)** | ***p* value (model)** | **Q (hetero-geneity)** | ***p* value (hetero-geneity)** | **Tau^2^** | **I^2^** | **R^2^** |
| --- | --- | --- | --- | --- | --- | --- | --- | --- | --- | --- |
| Low pollution intensity | -0.276 | -1.120 | 0.567 | 0.430 | 0.520 | 229.305 | < 0.001 | 4.709 | 95% |  |
| Extreme pollution intensity | -0.537 | -1.663 | 0.589 | 0.574 | 0.350 | 6.939 | 0.326 | 0.058 | 14% |  |
| Overall | -0.370 | -1.045 | 0.305 | 0.344 | 0.283 | 243.333 | < 0.001 | 3.027 | 93% | 0% |

| **Component of variance** | ***Q*** | ***d.f.*** | ***p*** |
| --- | --- | --- | --- |
| Low pollution intensity | 229.305 | 11 | < 0.001 |
| Extreme pollution intensity | 6.939 | 6 | 0.326 |
| Within | 236.244 | 17 | < 0.001 |
| Between | 0.131 | 1 | 0.717 |
| Total | 243.333 | 18 | < 0.001 |

**Supplementary Materials Table B.7–8** Estimates and heterogeneities in the models for Pb concentrations in ground beetle individuals living in uncontaminated and contaminated habitats

| **Subgroup** | **Mean effect size (model)** | **Lower CI bound (model)** | **Upper CI bound (model)** | **SE (model)** | ***p* value (model)** | **Q (hetero-geneity)** | ***p* value (hetero-geneity)** | **Tau^2^** | **I^2^** | **R^2^** |
| --- | --- | --- | --- | --- | --- | --- | --- | --- | --- | --- |
| Low pollution intensity | 0.154 | -0.544 | 0.853 | 0.356 | 0.665 | 55.047 | < 0.001 | 0.711 | 82% |  |
| Extreme pollution intensity | -1.584 | -2.322 | -0.846 | 0.377 | < 0.001 | 64.119 | < 0.001 | 2.435 | 83% |  |
| Overall | -0.667 | -1.174 | -0.159 | 0.259 | 0.010 | 139.129 | < 0.001 | 1.445 | 84% | 32.11% |

| **Component of variance** | ***Q*** | ***d.f.*** | ***p*** |
| --- | --- | --- | --- |
| Low pollution intensity | 55.047 | 10 | < 0.001 |
| Extreme pollution intensity | 64.119 | 11 | < 0.001 |
| Within | 119.166 | 21 | < 0.001 |
| Between | 11.238 | 1 | 0.001 |
| Total | 139.129 | 22 | < 0.001 |

**Supplementary Materials Table B.9–10** Estimates and heterogeneities in the models for Zn concentrations in ground beetle individuals living in uncontaminated and contaminated habitats

| **Subgroup** | **Mean effect size (model)** | **Lower CI bound (model)** | **Upper CI bound (model)** | **SE (model)** | ***p* value (model)** | **Q (hetero-geneity)** | ***p* value (hetero-geneity)** | **Tau^2^** | **I^2^** | **R^2^** |
| --- | --- | --- | --- | --- | --- | --- | --- | --- | --- | --- |
| Low pollution intensity | 0.341 | -0.291 | 0.973 | 0.323 | 0.290 | 381.806 | < 0.001 | 9.819 | 95% |  |
| High pollution intensity | -0.383 | -1.955 | 1.190 | 0.802 | 0.633 | 5.132 | 0.077 | 0.447 | 61% |  |
| Extreme pollution intensity | -0.559 | -1.216 | 0.098 | 0.335 | 0.095 | 36.960 | 0.002 | 0.000 | 57% |  |
| Overall | -0.114 | -0.552 | 0.323 | 0.223 | 0.609 | 532.901 | < 0.001 | 4.540 | 93% | 1.68% |

| **Component of variance** | ***Q*** | ***d.f.*** | ***p*** |
| --- | --- | --- | --- |
| Low pollution intensity | 381.806 | 18 | < 0.001 |
| High pollution intensity | 5.132 | 2 | 0.077 |
| Extreme pollution intensity | 36.9595 | 16 | 0.002 |
| Within | 423.898 | 36 | < 0.001 |
| Between | 3.865 | 2 | 0.145 |
| Total | 532.901 | 38 | < 0.001 |

**Supplementary Materials C.1** Funnel plot, regression tests for funnel plot asymmetry, and model results after trim and fill for Cd concentrations in ground beetle individuals living in uncontaminated and contaminated habitats


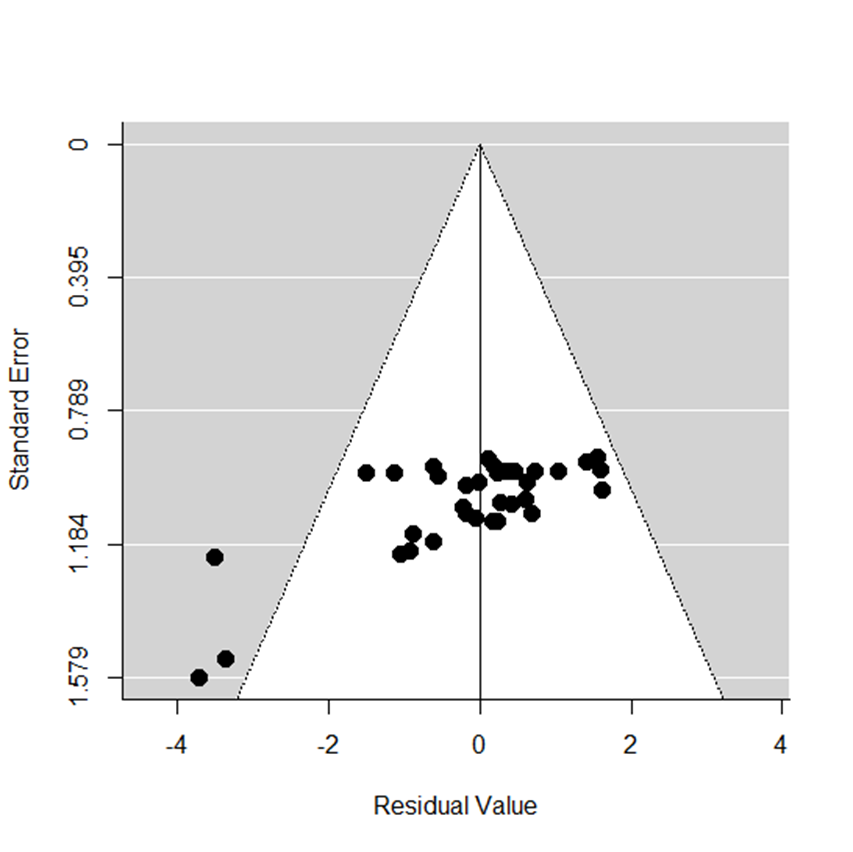


***Regression Test for Funnel Plot Asymmetry***

model: weighted regression with multiplicative dispersion

predictor: standard error

test for funnel plot asymmetry: t = -1.8702, df = 32, p = 0.0706

model: mixed-effects meta-regression model

predictor: standard error

test for funnel plot asymmetry: z = -3.9324, p < 0.0001

***Model Results after Trim and Fill:***

Estimated number of missing studies on the right side: 0 (SE = 3.4798)

| Hedge's *g* | SE | *z* value | *p* value | Lower CI bound | Upper CI bound |
| --- | --- | --- | --- | --- | --- |
| -1.1121 | 0.2036 | -5.4612 | < 0.001 | -1.5112 | -0.7130 |

**Supplementary Materials C.2** Funnel plot, regression tests for funnel plot asymmetry, and model results after trim and fill for Cu concentrations in ground beetle individuals living in uncontaminated and contaminated habitats


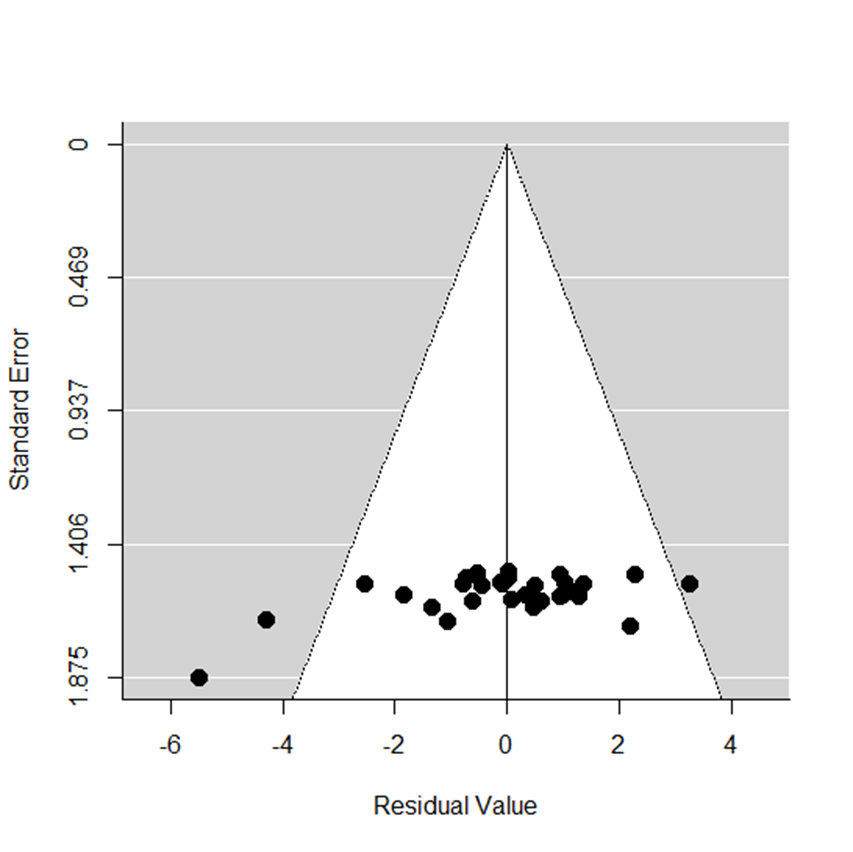


***Regression Test for Funnel Plot Asymmetry***

model: weighted regression with multiplicative dispersion

predictor: standard error

test for funnel plot asymmetry: t = -0.9423, df = 29, p = 0.3538

model: mixed-effects meta-regression model

predictor: standard error

test for funnel plot asymmetry: z = -2.4988, p = 0.0125

***Model Results after Trim and Fill:***

Estimated number of missing studies on the right side: 0 (SE = 3.0243)

| Hedge's *g* | SE | *z* value | *p* value | Lower CI bound | Upper CI bound |
| --- | --- | --- | --- | --- | --- |
| -0.1787 | 0.2898 | -0.6165 | 0.5376 | -0.7467 | 0.3893 |

**Supplementary Materials C.3** Funnel plot, regression tests for funnel plot asymmetry, and model results after trim and fill for Mn concentrations in ground beetle individuals living in uncontaminated and contaminated habitats


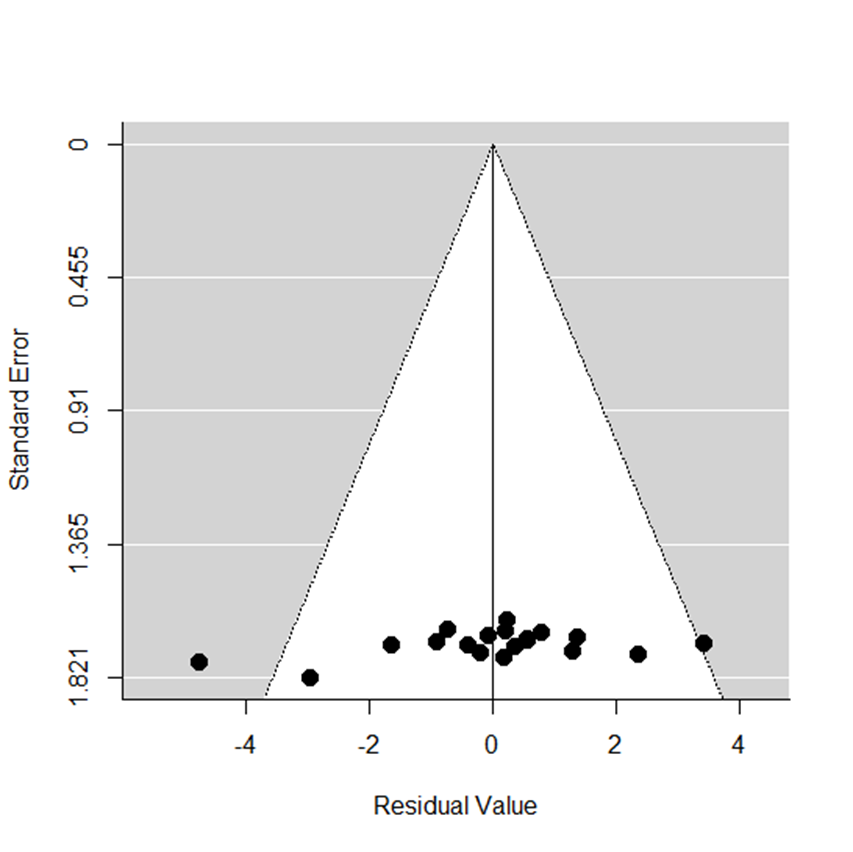


***Regression Test for Funnel Plot Asymmetry***

model: weighted regression with multiplicative dispersion

predictor: standard error

test for funnel plot asymmetry: t = -1.2067, df = 17, p = 0.2441

model: mixed-effects meta-regression model

predictor: standard error

test for funnel plot asymmetry: z = -1.7974, p = 0.0723

***Model Results after Trim and Fill:***

Estimated number of missing studies on the right side: 0 (SE = 2.5147)

| Hedge's *g* | SE | *z* value | *p* value | Lower CI bound | Upper CI bound |
| --- | --- | --- | --- | --- | --- |
| -0.3817 | 0.4029 | -0.9472 | 0.3435 | -1.1714 | 0.4081 |

**Supplementary Materials C.4** Funnel plot, regression tests for funnel plot asymmetry, and model results after trim and fill for Pb concentrations in ground beetle individuals living in uncontaminated and contaminated habitats


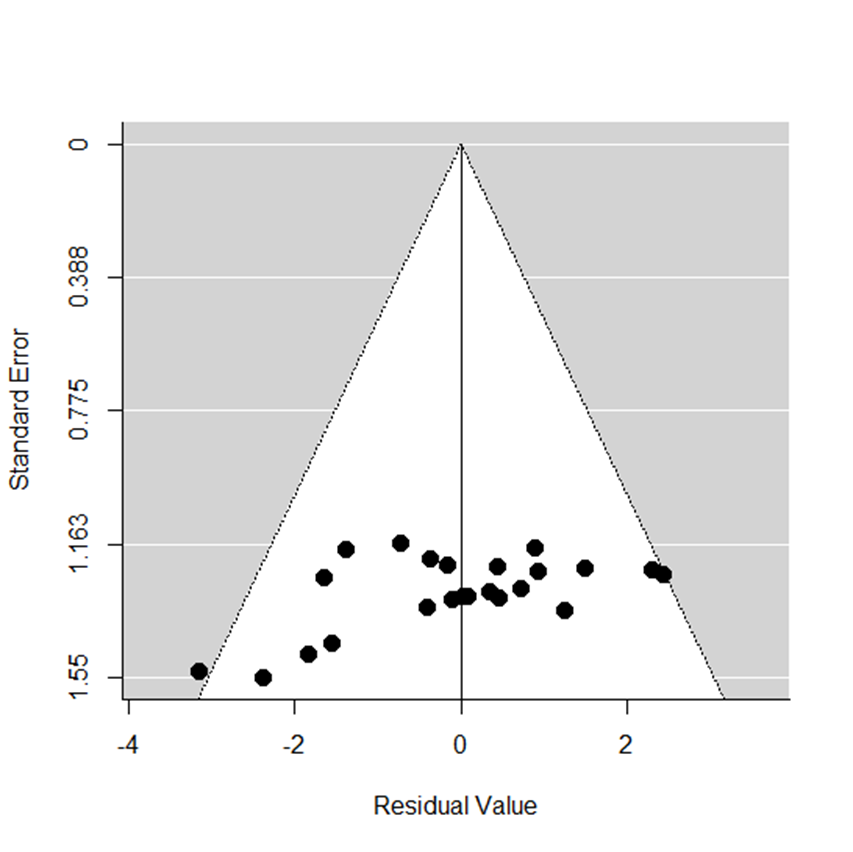


***Regression Test for Funnel Plot Asymmetry***

model: weighted regression with multiplicative dispersion

predictor: standard error

test for funnel plot asymmetry: t = -2.1138, df = 21, p = 0.0467

model: mixed-effects meta-regression model

predictor: standard error

test for funnel plot asymmetry: z = -4.0294, p < 0.0001

***Model Results after Trim and Fill:***

Estimated number of missing studies on the right side: 0 (SE = 2.9084)

| Hedge's *g* | SE | *z* value | *p* value | Lower CI bound | Upper CI bound |
| --- | --- | --- | --- | --- | --- |
| -0.7345 | 0.3311 | -2.2184 | 0.0265 | -1.3835 | -0.0856 |

**Supplementary Materials C.5** Funnel plot, regression tests for funnel plot asymmetry, and model results after trim and fill for Zn concentrations in ground beetle individuals living in uncontaminated and contaminated habitats


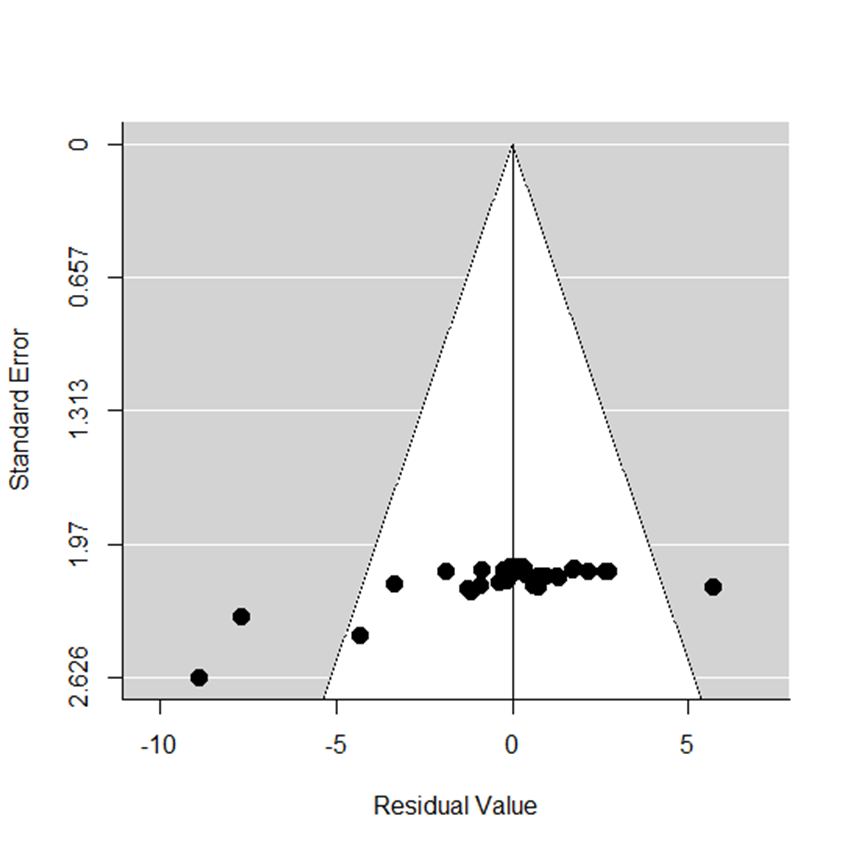


***Regression Test for Funnel Plot Asymmetry***

model: weighted regression with multiplicative dispersion

predictor: standard error

test for funnel plot asymmetry: t = -0.7883, df = 37, p = 0.4355

model: mixed-effects meta-regression model

predictor: standard error

test for funnel plot asymmetry: z = -4.6133, p < 0.0001

***Model Results after Trim and Fill:***

Estimated number of missing studies on the right side: 0 (SE = 3.5431)

| Hedge's *g* | SE | *z* value | *p* value | Lower CI bound | Upper CI bound |
| --- | --- | --- | --- | --- | --- |
| -0.2320 | 0.3557 | -0.6522 | 0.5143 | -0.9292 | 0.4652 |
